# Safe Resection of Renal Cell Carcinoma with Liver Invasion Using Liver Hanging Technique Supported by Preoperative Portal Vein Embolization

**DOI:** 10.1155/2018/5139034

**Published:** 2018-06-28

**Authors:** Masato Fujii, Toshio Kamimura, Hiromasa Tsukino, Eiji Furukoji, Tatefumi Sakae, Koichi Yano, Naoya Imamura, Shoichiro Mukai, Atsushi Nanashima, Toshiyuki Kamoto

**Affiliations:** ^1^Department of Urology, Faculty of Medicine, University of Miyazaki, Miyazaki, Japan; ^2^Department of Radiology, Faculty of medicine, University of Miyazaki, Miyazaki, Japan; ^3^Department of Surgery, Faculty of Medicine, University of Miyazaki, Miyazaki, Japan

## Abstract

In cases of RCC with liver involvement, partial hepatectomy is known to provide a better chance of survival for patients. For this reason, complete resection with clear surgical margin is thought to be necessary to achieve favorable outcome. Anterior liver hanging maneuver was extremely useful during hemihepatectomy in this rare type of RCC. A 63-year-old male was diagnosed with a large right renal cell carcinoma. The tumor measured 10 cm in diameter with tumor thrombus toward the inferior vena cava (IVC). In addition, we observed direct infiltration to the liver. We attempted a preoperative portal vein embolization (PVE) to preserve residual liver volume and function after right lobectomy. After PVE the resected volume decreased from 921 cm^3^ (71%) to 599 cm^3^ (53.4%). During the procedure, a nasogastric tube was placed in the retrohepatic space for liver hanging maneuver according to the original Belghiti's maneuver after dissection of the renal artery and vein. After hepatic parenchymal transection exposing vena cava, the right hepatic veins were safely transected using vascular stapler; right nephrectomy and hemihepatectomy were performed. The patient recovered without postoperative hepatic or urinary complications and has remained free of local recurrence and any de novo metastasis for 18 months.

## 1. Introduction

Approximately 20-30% of patients with renal cell carcinoma (RCC) are reported to have metastasis at the time of diagnosis and distant metastasis after surgical intervention for primary tumor [1]. Indication for metastatic RCC (mRCC) patients remains controversial. Conti et al. reported that median survival among patients having received cytoreductive nephrectomy improved from 13 to 19 months in the era of targeted therapy, while survival among patients not receiving cytoreductive nephrectomy increased slightly (from 3 to 4 months) [2]. On the other hand, surgical intervention is performed for locally advanced RCC. For RCC involving adjacent organs, en bloc removal of kidney and involved organ is required for cancer control. In cases of liver involvement, partial hepatectomy provides a better chance of survival; therefore, complete resection with clear surgical margin is necessary to achieve favorable outcome. However, in case of high-volume major hepatectomy, the rate of liver failure is reported to be relatively high in the absence of preoperative manipulation to preserve liver volume and function [3]. In particular, major hepatectomy after multidrug chemotherapy for longer periods led to high risk of posthepatectomy morbidity and mortality in the case of liver metastases originating from colorectal carcinoma [4]. Preoperative portal vein embolization (PVE) is an ideal radiological intervention inducing hypertrophy of remnant liver to avoid postoperative hepatic insufficiency [5]. This two-step perioperative strategy of PVE and major hepatectomy is necessary in the case of combined resection with right nephrectomy and neoadjuvant chemotherapy for large RCC as well. Although the mobilization of the lateral side of the right liver is a standard procedure, it is difficult to mobilize in the case that large RCC is involved and the right liver is lifted toward the ventral abdominal wall or diaphragm. An alternative safe approach for right hepatectomy with nephrectomy is, therefore, necessary to avoid the operative risk of massive bleeding. The anterior approach applying liver hanging maneuver (LHM) has been reported to be a useful option for such cases [6].

In the present report, we experienced a rare case of advanced stage RCC with direct hepatic invasion. We herein report that a well-planned collaborative surgery with liver surgeons was successfully performed by combining the latest neoadjuvant chemotherapy, the preoperative PVE, and the anterior approach using LHM.

## 2. Case Presentation

**Figure 1:**
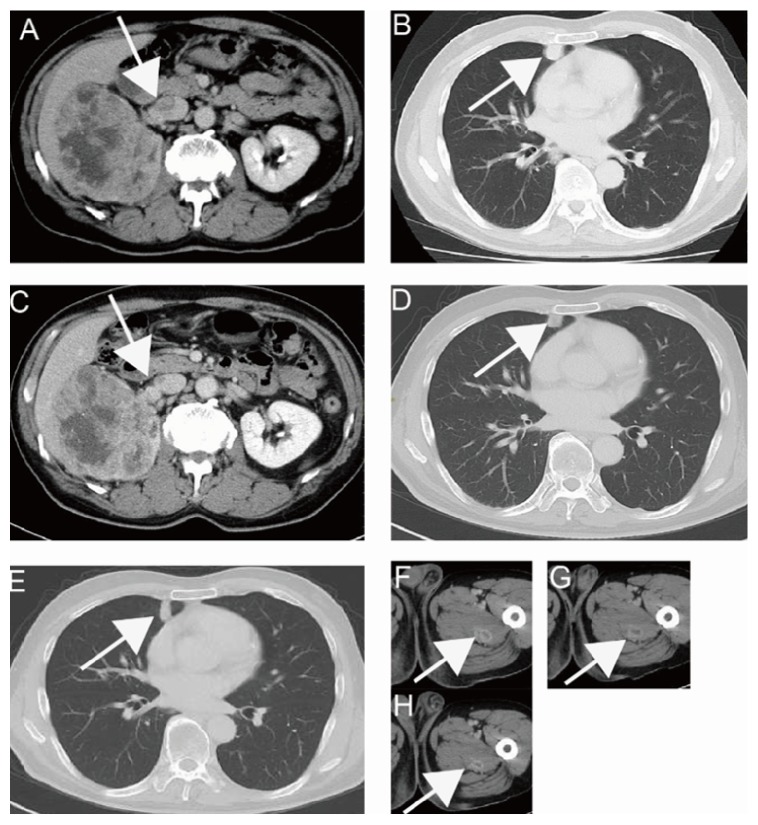
*Computed tomography (CT) finding of primary site (A, C), lung metastasis (B, D, E), and intramuscular metastasis (F-H)*. Hypervascular renal tumor with liver invasion, IVC extension (arrowhead, left), and lung metastasis (arrowhead, right) were observed. After a month of presurgical treatment, tumor thrombus and lung metastasis had decreased (A-B, F: before treatment, C-D, G: after treatment). CT appearance of lung metastasis and that of intramuscular metastasis at 18 months after surgery are shown (E, H).

**Figure 2:**
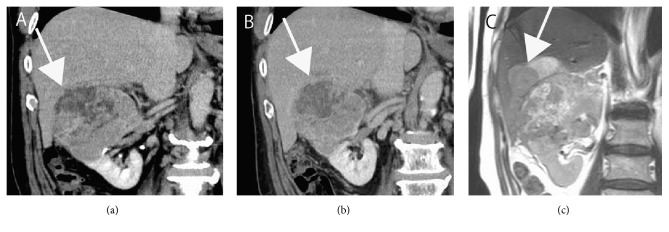
*CT and magnetic resonance imaging (MRI) findings at the site of liver invasion*. Appearance of invasion front (arrows) is shown before axitinib treatment (a), 2 months after treatment (b), and 3 months after treatment (c). Focal invasion is suspected at pretreatment period (a); however, invasion was progressed at 2 months after treatment (b), and apparent nodular formation was observed at 3 months (c).

A 63-year-old male presented to a private hospital complaining of asymptomatic gross hematuria. Computed tomography (CT) showed a hypervascular tumor affecting the right kidney. The tumor measured 10 cm in diameter with tumor thrombus toward the inferior vena cava (IVC) (Figure 1(A)). In addition, direct infiltration to the liver was observed (Figure 2(a)). Regional lymph node metastasis, multiple lung metastasis (Figure 1(B)), and intramuscular metastasis of left femoral muscle (Figure 1(F)) were also observed (clinical staging of T4N1M1). The patient was referred to our hospital for treatment. Initially, indication of cytoreductive nephrectomy was questionable; therefore, we administered presurgical axitinib treatment according to our previously described protocol [7]. One-month treatment achieved shortened tumor thrombus and shrinkage of the primary site (Figure 1(C)); however, liver invasion had progressed (Figure 2(b)). Lung and intramuscular metastases were controllable (Figures 1(D) and 1(G)). In spite of an increase in the dose of axitinib, liver infiltration was revealed to be worsening at 2 months from initial treatment (Figure 2(c)). Therefore, we considered immediate surgical intervention with en bloc right nephrectomy and hemihepatectomy. After discussion with liver surgeons, we attempted a perioperative PVE to preserve residual liver volume and function after right lobectomy (including invaded tumor) in consideration of chemotherapy-induced liver functional deterioration and high risk of major hepatectomy.

**Figure 3:**
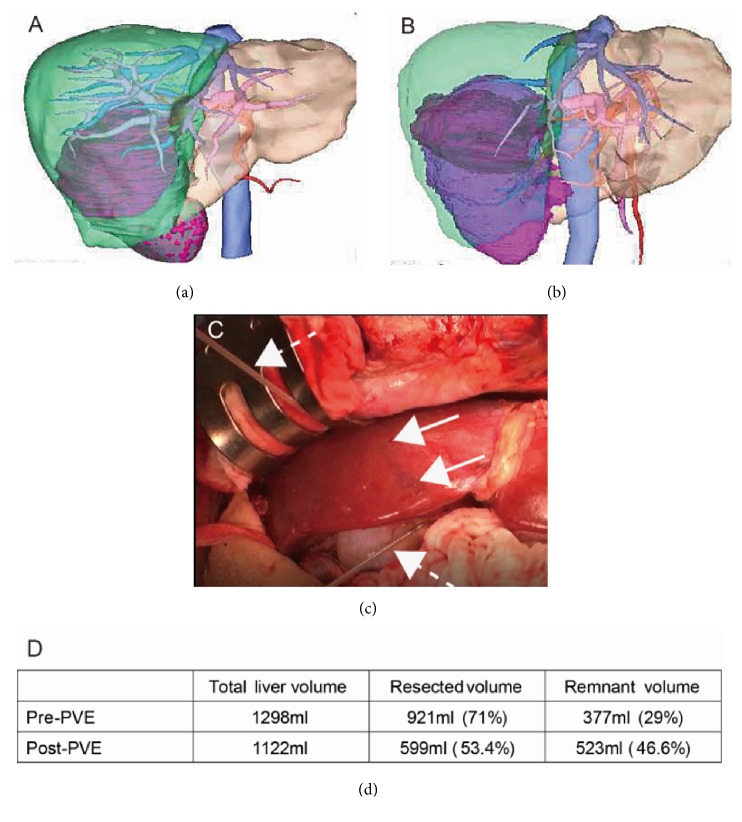
*Result of volumetric analysis by the Synapse Vincent Work Station ((a) before portal vein embolization (PVE), (b) after PVE, D: estimated liver volume) and intraoperative finding of the liver (c)*. In 3-dimensional graphics of (a) and (b), estimated resected liver is shown in green, estimated remnant liver is light brown, IVC and major veins are blue, portal vein is pink, and kidney is purple. The estimated resected tumor liver volume was 921 ml (71% of whole liver) at pre-PVE status (a). After PVE, estimated resected liver volume reduced to 599 ml (53.4% of whole liver) (b). Intraoperative findings showed morphologically shrunken right liver parenchyma (arrows show the midline) due to portal ischemia (c). Hanging tube is shown by dotted arrows. Total and estimated liver volume are shown (d).

Department of Surgery policy at our institute requires that indocyanine green retention rate at 15 minutes (ICGR15) be determined preoperatively for the liver to be resected using the formula described by Takasaki et al. [8]. The estimated resected liver volume, excluding tumor volume (cm^3^), is measured by computed tomography volumetry [9]. The present volumetric analysis was conducted using Synapse Vincent Work Station (Fujifilm Medical Co., Tokyo, Japan). Essentially, in cases where the permitted resected volume is less than the estimated volume, or the estimated volume is greater than 65% in normal liver, preoperative PVE is selected [10]. In the present case, ICGR15 was 5.7% and the comprehensive evaluation of liver function was Child-Pugh grade A. The estimated resected liver volume was 921 cm^3^ (71% of the whole liver) (Figure 3(a)). PVE was performed by 2 interventional radiologists. Substances used for embolization were 4 sheets of gelatin lipiodol, Serescue (Nihon-Kayaku Co., Tokyo, Japan), mixed in contrast media, and 2 permanent microcoils were subsequently placed in the right portal veins. The post-PVE coarse was uneventful, resected volume was reduced from 921 cm^3^ to 599 cm^3^ (53.4 % of the whole liver), and an increase in remnant left liver volume of 523 cm^3^ (46.6 %) was achieved on day 14 after PVE (Figures 3(b) and 3(d)). Preoperative ICGR15 was mildly worsened at 18%; however, the permitted resected volume by Takasaki's formula was maintained. The scheduled operation was performed on day 35 after PVE following a 3-day drug-off period.

The patient was placed in the left hemilateral position. Thoracoabdominal incision was made via the 9th intercostal space accompanied by upper abdominal midline incision. The urological surgeons first mobilized the ascending, transverse colon, and duodenum, and the right renal artery was dissected at interaortocaval region. Intraoperative ultrasound examination revealed that a shortened tumor thrombus remained within the renal vein and right renal vein dissection. Because of the tight connection between the kidney and liver and confirmation of tumor connection to the right side of vena cava, we were unable to perform ordinary liver mobilization at this stage. At this point liver surgeons were deployed. Placement of nasogastric tube in the retrohepatic space for LHM was performed according to the original Belghiti's maneuver (Figure 3(c)) [10]. Prior to the transection, the right hepatic artery and portal vein were ligated and divided after confirmation of tumor invasion into the liver. Liver transection at the midline of the liver was easily achieved under intermittent hepatic inflow occlusion (three sessions at 15-minute intervals) and continuous hemiclamp of the infrahepatic vena cava through the maintenance of central venous pressure at 8 mmHg. After hepatic parenchymal transection exposing vena cava, the right hepatic veins were safely transected using vascular stapler. Finally, urological surgeons performed partial resection with direct closure of vena cava infiltrated by the tumor under good operative view and eventually achieved combined resection of right kidney and right liver. Apparent invasion to other retroperitoneal tissue was not observed except for right adrenal gland. Total operation time was 8 hours and 47 minutes, and total bleeding volume was 2370 ml. The patient recovered without postoperative hepatic or urinary complications and has remained free of local recurrence and any de novo metastasis for 18 months (Figures 1(E) and 1(H)). Tyrosine kinase inhibitor treatment was initiated one month after surgery.

### 2.1. Pathological Findings

**Figure 4:**
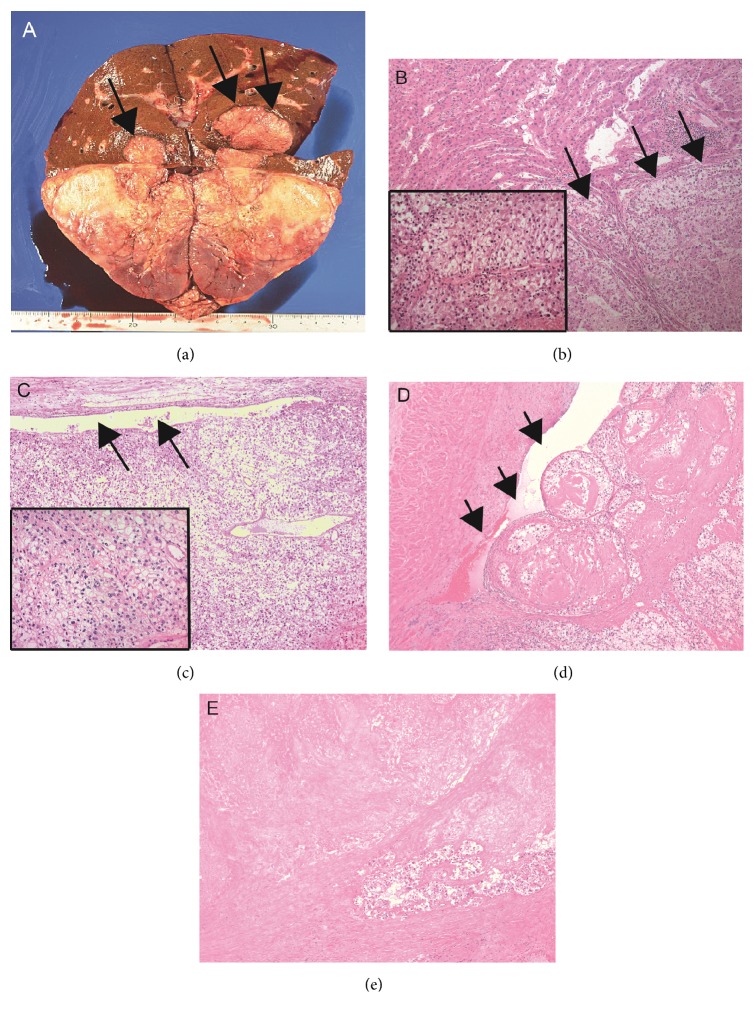
*Macroscopic appearance (a) and pathological findings (b-e) of resected specimen*. Renal tumor directly invading the right lobe of the liver (arrows, (a)). Tumor cells showing clear cytoplasm with atypical nuclei infiltrated into the liver parenchyma ((b) invasion front is shown by arrows). Pathological appearance of tumor cells at invasion front is compatible with conventionally clear cell-type renal cell carcinoma (inset, high magnification). The primary tumor also shows similar pathological findings ((c) arrows show renal pelvis, inset: high magnification). Infiltration to large vessel ((d) arrows) and large amount of necrotic area (e) are observed.

The tumor was composed of atypical polygonal cells (Fuhrman grade 2) with clear cytoplasm proliferating mainly in solid or nested fashion (Figure 4(c)), and papillary architecture was also observed in part of the tumor. The pathological findings were compatible with clear cell RCC. The tumor cells infiltrated directly to the renal vein, renal pelvis, right adrenal gland, and the liver (Figures 4(a)-4(b)). Apparent pathological difference between primary site and invasion front was not confirmed (Figure 4). Necrosis was observed in approximately 50% of the tumor with organization of obstructed medium-sized vessels, suggesting the effect of presurgical treatment (Figure 4). Apparent infiltration of cancer cells to vena cava was not observed.

## 3. Discussion and Conclusions

### 3.1. PVE

Kinoshita et al. introduced preoperative portal vein embolization to prevent posthepatectomy liver insufficiency [11]. The basic principle involved occluding a branch of portal flow, which subsequently led to ipsilateral hepatic atrophy and compensatory contralateral hypertrophy. Makuuchi et al. first introduced this concept to routine clinical practice in patients with cholestatic liver disease, chronic hepatitis, or cirrhosis to increase the number of patients suitable for curative surgery [12]. The safety of PVE has been clarified and the indication for PVE has been extended to any major resection requiring preoperative manipulation to increase liver volume [13]. Indeed, PVE has been reported to reduce the risk of postoperative liver failure after partial hepatectomy in many cases [14], and the actual functional changes to remnant liver seem to be higher in comparison with morphological volumetric changes [15]. In the current case, PVE procedure was performed under local anesthesia. The left branch of portal vein was punctured with a18G/15 cm percutaneous transhepatic cholangiodrainage (PTCD) puncture needle (CX-PTC needle, Gadelius Medical K. K., USA) using ultrasound. After successful portal vein puncture, microcatheter (Carnelian® ER, TOKAI MEDICAL PRODUCTS, Japan) and Microguidewire (FATHOM™-16, Boston Scientific, USA) were placed in the portal vein through the outer plastic tube of the PTC needle. After insertion of microcatheter into the right portal vein, embolization was performed by injection of gelatin sponge (Serescue®, Nihon-Kayaku Co., Japan) gel with iodized oil (Lipiodol®, Guerbet, France) and 2 microcoils. As a result, proposed liver remnant increased from 30% to 43.9% during a one-month period. In addition, favorable remnant liver function was preserved after surgery. To the best to our knowledge, this case is the first report of PVE indicated to RCC in English literature.

### 3.2. Liver Hanging Maneuver

Combined surgery of nephrectomy and right lobectomy is required for cases with locally advanced RCC that has directly infiltrated into the right lobe of the liver. Conventionally, the right liver is mobilized completely during this surgery; however, this technique is preferably applied for cases in which hepatic mobilization is risky or difficult, cases such as a large liver tumor, tumor invading surrounding vessels or organs. In our case, a large renal tumor was tightly connected to the right liver, the surrounding retroperitoneal tissue, and vena cava, and lifting space could not be obtained due to limitation created by the costal arch; therefore, conventional mobilization could not be performed. For the combined resection of the kidney and liver, we chose an anterior approach using liver hanging maneuver (an 8Fr nasogastric tube was used for liver suspension), as reported by Belghiti et al. [6]. As a result, en bloc resection was successfully performed without severe intraoperative complications. This report is the fourth case of resection of large RCC by anterior approach using liver hanging maneuver [16].

### 3.3. Indication for Cytoreductive Nephrectomy for Patients with mRCC in the Era of TKI

The prognosis of mRCC patients is reported to be poor (5-year survival rates do not exceed 30%) in spite of improved agents, including TKI or immune checkpoint inhibitors over the last decade [17]. Indication for mRCC patients remains controversial. Conti et al. reported that median survival among patients having received cytoreductive nephrectomy improved from 13 to 19 months in the era of targeted therapy, while survival among patients not receiving cytoreductive nephrectomy increased slightly (from 3 to 4 months) [2]. Another study suggested that young male patients with oligometastases and good performance status might benefit from cytoreductive surgery [18]. However all studies were retrospective, and we await the result of an ongoing prospective study. In our case, primary tumor and metastatic sites were decreased and tumor thrombus was shortened by presurgical axitinib treatment; however, the size of intrahepatic section (invasion front) of the RCC was increased. Discrepancy of treatment effect is sometimes observed in patients with mRCC. Intratumor heterogeneity in RCC has been reported [19]. In addition, the microenvironment around tumor cells may protect against treatment as a so-called sanctuary site for tumor cells. For such cases, surgical intervention is the only option for cancer control. In our case, postoperative sorafenib treatment maintained stable disease for 6 months after surgery without any de novo metastases or local recurrence.

We successfully performed en bloc resection of right kidney with major hepatectomy for locally advanced RCC by applying precise preoperative preparations as the latest neoadjuvant chemotherapy and effective PVE, and safe operative skills in collaboration with urological and hepatic surgeons and radiologists. A well-considered plan created to ensure the inclusion of integrated preoperative and intraoperative expertise is required to promote successful outcomes for advanced stage patients.

## Abbreviations

PVE: Perioperative portal vein embolization
RCC: Renal cell carcinoma
IVC: Inferior vena cava
LVH: Liver hanging maneuver
CT: Computed tomography
ICGR: Indocyanine green retention rate.
